# From Competence to Character: Reimagining Neurology Education for a Complex and Changing World

**DOI:** 10.1212/NE9.0000000000200251

**Published:** 2025-09-09

**Authors:** Roy Strowd

**Affiliations:** Department of Neurology, Wake Forest University School of Medicine, Winston Salem, NC.

This issue of *Neurology*®* Education* presents a diverse collection of scholarship. From telestroke and simulation-based instruction to functional neurologic disorders and climate change education, the articles in this issue address both emerging and enduring themes in the field. Together, the articles highlight a growing shift in neurology training, one that prioritizes not only technical competence but also development of dispositions and the broader societal roles neurologists are called to fulfill.

## Reclaiming Character Within the Hidden Curriculum: Dispositional Learning

Dispositional learning is a vital yet often overlooked component of professional identity formation in neurology. Dispositional learning refers to educational activities that shape the habits of mind, attitudes, and character traits. This includes the cognitive, emotional, and behavioral tendencies that influence how learners approach uncertainty, connect empathically, and define their clinical roles. Traits such as curiosity, intellectual humility, perseverance, empathy, and compassion profoundly affect how students engage with patients, pursue lifelong learning, and navigate complexity. These habits can be cultivated and awareness of dispositions taught.

Unlike knowledge or procedural skill, dispositional learning focuses on who learners are becoming, not just what they can do. This is especially critical in neurology, where ambiguity and ethical nuance are common. Dispositional objectives can and should be assessed. In an age where medical knowledge is widely accessible to all through the click of a button, cultivating reflective, compassionate, and adaptive clinicians is more important than ever.^1^

Several articles in this issue underscore the need to explicitly address character-based dimensions of training in neurology. In “From Brain to Being: Reintegrating Philosophy Into Neurology Education,” the authors offer strategies for incorporating philosophy of mind into graduate medical education to enhance diagnostic reasoning and patient-centered care.^2^ Short et al.^3^ document the prevalence of secondary trauma in students on the neurology clerkship who are exposed to emotionally complex cases, highlighting the need for emotional resilience training. Another gap analysis study reveals ongoing stigma and outdated attitudes toward functional neurologic disorders, pointing to the need to develop empathy and tolerance for diagnostic uncertainty.^4^

If dispositional learning is fostered through reflective practice, role modeling, ethics discussions, simulation, and longitudinal relationships, this issue invites us to imagine how character education, narrative medicine, and the medical humanities might fill long-standing gaps in neurology education.^5^ This can be incorporated into existing curricula, but more work is needed to demonstrate successful pilot programs in neuroscience instruction.^6^

## Teaching Human Intelligence Within Technology-Enabled Learning Systems

As clinical care becomes increasingly shaped by artificial intelligence (AI), neurology education must also evolve to teach learners how to think both *about* and *with* AI. Mastery now includes not only understanding large language models (LLMs) and AI-based diagnostic tools but also preserving human connection as central to effective, ethical care.

In their article on shared simulation, Harrison et al.^7^ show how repurposed simulation cases can be effectively adapted across learner groups providing a model for scalable, real-world simulation training. Soon AI-enabled simulation will allow for even more rapid customization of training experiences. LLMs already support case creation, simulation checklists, and assessment design.^8-10^ AI-generated e-learning allows learners to personalize their learning and practice different iterations of a patient scenario from the comfort of their living room. As digital tools proliferate, curricula must increasingly emphasize the uniquely human capacities that AI cannot replicate–empathy, communication, adaptability, and personal connection.

## Preparing for Purpose: Advocacy and Community Engagement

Neurology education must also equip learners to engage with society. Societal responsibility extends beyond individual patient care to addressing the broader determinants of health, including climate change, planetary health, and environmental justice. Growing evidence links environmental factors to neurologic disease, positioning neurologists to play a critical role in understanding and addressing these interdependencies.

In “Integrating Climate Change Into Neurology Education: Preparing Future Physicians for a Warming World,” the authors offer concrete strategies for embedding climate-related content into neuroscience instruction.^11^ Learners are trained to recognize climate-modulated illnesses, advocate for vulnerable populations, and understand their responsibility as clinicians in a warming world. Climate-relevant competencies lie at the intersection of neuroscience, policy, and ethics. But they have yet to be universally defined. Integrating these themes into training may be essential for preparing neurologists to lead amid a changing world but before this occurs, we need a clear goal in mind. What does it mean to be a climate-educated neurologist? How do we know when this has been achieved and what are the appropriate outcomes that will reliably track progress towards mastery? The societal imperative is strong but much work remains to understand how appropriate climate-informed education can be reliably implemented in the field.

## A Call for an Expanded Vision of Neurology Education

Taken together, the articles in this issue argue for an expanded educational vision for neuroscience education, one that integrates competence, character, and community.

1. Competence encompasses the technical skills, diagnostic reasoning, and clinical mastery our patients expect.
2. Character involves the internal dispositions, empathy, humility, and professional integrity that shape who we are as physicians.
3. Community engagement leverages our responsibility as advocates, stewards, and system thinkers.

This vision invites us to reframe our teaching not only around what our learners know and do, but who they become and what they are empowered to change.

Reimagining *Neurology® Education* begins here. Enjoy your reading.
